# Intracameral Bevacizumab Versus Sub-Tenon’s Mitomycin C as Adjuncts to Trabeculectomy: 3-Year Results of a Prospective Randomized Study

**DOI:** 10.3390/jcm10102054

**Published:** 2021-05-11

**Authors:** Gerasimos Kopsinis, Dimitrios Tsoukanas, Dimitra Kopsini, Theodoros Filippopoulos

**Affiliations:** 1 Glaucoma Service, Athens Vision Eye Institute, 328-330 Siggrou Ave, 17673 Athens, Greece; gkopsinis@gmail.com (G.K.); tfilippopoulos@gmail.com (T.F.); 2 Comprehensive Clinical Trials Unit, University College London, 90 High Holborn, London WC1V 6LJ, UK; d.kopsini@gmail.com

**Keywords:** trabeculectomy, bevacizumab, mitomycin C, filtration surgery, anti-fibrotic agents, wound healing

## Abstract

Conjunctival wound healing determines success after filtration surgery and the quest for better antifibrotic agents remains active. This study compares intracameral bevacizumab to sub-Tenon’s mitomycin C (MMC) in trabeculectomy. Primary open-angle or exfoliative glaucoma patients were randomized to either bevacizumab (*n* = 50 eyes) or MMC (*n* = 50 eyes). The primary outcome measure was complete success, defined as Intraocular Pressure (IOP) > 5 mmHg and ≤21 mmHg with a minimum 20% reduction from baseline without medications. Average IOP and glaucoma medications decreased significantly in both groups at all follow-up points compared to baseline (*p* < 0.001), without significant difference between groups at 3 years (IOP: bevacizumab group from 29 ± 9.4 to 15 ± 3.4 mmHg, MMC group from 28.3 ± 8.7 to 15.4 ± 3.8 mmHg, *p* = 0.60; Medications: bevacizumab group from 3.5 ± 0.9 to 0.5 ± 1, MMC group from 3.6 ± 0.7 to 0.6 ± 1.1, *p* = 0.70). Complete success, although similar between groups at 3 years (66% vs. 64%), was significantly higher for bevacizumab at months 6 and 12 (96% vs. 82%, *p* = 0.03; 88% vs. 72%, *p* = 0.04, respectively) with fewer patients requiring medications at months 6, 9 and 12 (4% vs. 18%, *p* = 0.03; 6% vs. 20%, *p* = 0.04; 8% vs. 24%, *p* = 0.03, respectively). Complication rates were similar between groups. In conclusion, intracameral bevacizumab appears to provide similar long-term efficacy and safety results as sub-Tenon’s MMC after trabeculectomy.

## 1. Introduction

For the past few decades, guarded trabeculectomy with antimetabolites has been, and still remains, the most popular surgical procedure for glaucoma treatment [1,2,3]. This continues to be the case despite the variety and magnitude of complications associated with the procedure [4,5].

The main reason for trabeculectomy failure is subconjunctival scarring at the site of the filtering bleb due to inflammation, angiogenesis and progressive fibrosis [6,7]. Therefore, conjunctival wound healing modulation is critical for success after glaucoma filtration surgery. Although mitomycin C (MMC) and 5-fluorouracil (5-FU) have improved trabeculectomy outcomes, they have been associated with increased incidence and severity of complications [8,9]. Furthermore, surgery can fail despite the use of antimetabolites [10]. Hence, we still need to find agents with better efficacy and safety profiles.

Vascular endothelial growth factor (VEGF) plays an important role in wound healing by inducing fibroblast proliferation and vascular permeability [11,12]. Studies have demonstrated that VEGF is higher in the aqueous humor and Tenon’s capsule of patients with glaucoma, that VEGF levels increase as IOP increases and that surgical success is adversely affected by higher preoperative VEGF levels. Moreover, administration of anti-VEGF agents, such as bevacizumab, downregulates fibroblast proliferation in vitro and reduces scar formation when injected at the site of trabeculectomy in rabbits [13,14].

The use of anti-VEGF agents as adjuncts to trabeculectomy via different routes of administration has been under investigation. Two systematic reviews of published randomized controlled trials concluded that, although subconjunctival bevacizumab improved surgical success, it increased the risk of early bleb leaks when compared to placebo [15,16]. Additionally, when compared to MMC, its effect on IOP control remained uncertain, as it seemed to increase the rate of encapsulated blebs. A randomized pilot study showed that combination of intravitreal ranibizumab with MMC resulted in better bleb morphology than MMC alone [17]. The effect of intracameral bevacizumab during trabeculectomy has only been addressed by three randomized studies. The first compared bevacizumab to placebo and concluded that bevacizumab significantly reduces the need for additional interventions during the first postoperative year [18]. The other two, conducted by the same group, compared bevacizumab to placebo and MMC, respectively, and showed that even though bevacizumab is effective in lowering IOP, it increases early bleb leakage. These studies reported on a mean follow-up period of 11 and 17 months, respectively [19,20].

This study aims to add to the pre-existing knowledge on this matter by providing longer follow-up, i.e., a minimum of three years and, to help clarify whether trabeculectomy with a single intracameral injection of bevacizumab alone is safe and effective, compared to standard guarded trabeculectomy with sub-Tenon’s MMC.

## 2. Materials and Methods

### 2.1. Design

This prospective, two-arm, parallel, randomized, open-label comparative study was performed at the Athens Vision Eye Institute, a tertiary referral eye center in Athens, Greece. Patients were randomized (simple randomization) to undergo either standard guarded trabeculectomy with sub-Tenon’s MMC, ‘the MMC group’ or standard guarded trabeculectomy with intracameral bevacizumab, ‘the bevacizumab group’. This study has been registered on clinicaltrials.gov (Identifier: NCT02901236).

### 2.2. Population

Consecutive patients with medically uncontrolled glaucoma or intolerance to glaucoma medications, who were evaluated at the glaucoma service of the Athens Vision Eye Institute and were scheduled for primary filtration surgery between January 2012 and May 2015, were assessed for eligibility. Adult patients with primary open-angle glaucoma (POAG) or exfoliative glaucoma (XFG) with either documented progressive disease or IOP above target on maximal tolerated medical treatment on at least 2 different occasions, who had the ability to attend regular follow-up visits, were included. For patients eligible for surgery in both eyes, only the first eye undergoing surgery was included in the study. This has been almost exclusively the eye with the worst perimetric loss at presentation. Exclusion criteria included: all other types of glaucoma, a history of ocular trauma or previous intraocular surgery other than uncomplicated clear-corneal phacoemulsification surgery, the need for combined phacotrabeculectomy, any prior anti-VEGF treatment and a known allergy to bevacizumab or mitomycin C. Patients who were pregnant, breast feeding or had a history of a systemic thromboembolic event during the last 6 months before surgery were also excluded.

### 2.3. Preoperative Evaluation

Before surgery, all patients underwent a comprehensive eye exam including slit lamp biomicroscopy (Takagi Seiko Co., Nakano City, Japan), determination of Snellen best corrected visual acuity (BCVA) (Reichert Inc., Depew, NY, USA), Goldmann applanation tonometry (GAT) (Takagi Seiko Co., Nakano City, Japan), on 2 separate occasions at least one week apart, gonioscopy (Ocular Instruments Inc., Bellevue, WA, USA), and dilated fundus examination. All patients had at least one visual field test on file, performed not more than 6 months prior to randomization. The Humphrey Visual Field Analyzer (HFA II-i, Carl Zeiss Meditec Inc., Dublin, CA, USA) with the 24/2 SITA-Standard protocol was used in all instances. Recorded data included age, race, gender, diagnosis, study eye, number and type of glaucoma medications, IOP, BCVA, central corneal thickness (CCT), cup to disc ratio and lens status.

### 2.4. Surgical Technique

Surgery was performed under topical anesthesia by a single surgeon (G.K.) using identical surgical technique except for the type of antimetabolite/antifibrotic agent employed. Patients were prepped and draped in a usual sterile way. A fornix-based conjunctival peritomy was performed with wide blunt dissection extending posteriorly. Wet-field cautery was utilized to achieve hemostasis. A 4 mm × 4 mm partial thickness scleral flap was dissected extending through the limbus into the cornea. In the MMC group, MMC 0.02% (Kyowa Kirin Ltd., Galashiels, UK) was applied using multiple soaked sponges under Tenon’s capsule for 2 min, before creating the scleral flap. The sponges were removed, and the surgical area was copiously irrigated with balanced salt solution (BSS; Alcon Laboratories Inc., Fort Worth, TX, USA). A paracentesis was created to establish access to the anterior chamber. The scleral flap was secured with two preplaced 8-0 Vicryl sutures (Ethicon, Inc., Somerville, NJ, USA). Subsequently, the anterior chamber was entered under the scleral flap and trabeculectomy was performed utilizing a Kelly Descemet punch (0.5 mm) (Storz, Heidelberg, Germany) followed by a surgical iridectomy using Vannas scissors (World Precision Instruments, Sarasota, FL, USA). The anterior chamber was re-inflated with BSS and a slow egress of aqueous without collapse of the anterior chamber was confirmed. Finally, the conjunctiva was closed with a running 10-0 Nylon suture (Alcon Laboratories Inc., Fort Worth, TX, USA). The anterior chamber was formed with BSS and the incisions were examined for leaks. In the bevacizumab group, trabeculectomy was performed identically, but without applying MMC. At the end of the case, 1.25 mg of bevacizumab (Avastin; Genentech, San Francisco, CA, USA) was injected into the anterior chamber through the paracentesis. Post-operative management constituted of topical ciprofloxacin (Cooper S.A., Athens, Greece) (every 3 h while awake for 2 weeks) and topical dexamethasone 0.1% (Cooper S.A., Athens, Greece) (every 3 h while awake for 2 weeks followed by a slow taper over the next 6 weeks).

### 2.5. Postoperative Evaluations

All patients were examined on the first day after surgery and then every week for the first month, every 3 months for the first year and every 6 months thereafter, until they reached 36 months of follow-up. Data from weeks 2 and 3 are not presented in this paper as they do not add value to the analysis. During each visit, a comprehensive ophthalmic examination was carried out. Collected data included IOP, glaucoma medication requirements, BCVA, early and late complications and additional surgical interventions.

### 2.6. Outcome Measures

Complete success, i.e., IOP ≤ 21 mmHg and > 5 mmHg along with at least 20% IOP reduction from baseline without medications based on Tube Versus Trabeculectomy (TVT) study criteria [21], was set as the primary outcome measure. Qualified success was the secondary outcome measure and was defined as above but with the addition of glaucoma medications. Patients who required de novo glaucoma surgery during the follow-up period were considered as surgical failures and did not contribute data on IOP or medications thereafter. Interventions at the slit lamp such as 5-FU (Bausch Health Companies Inc., Laval, QC, Canada) injections and bleb needling procedures did not qualify as failures, but were recorded along with postoperative complication rates.

### 2.7. Randomization and Blinding (Masking)

Simple randomization was used to assign patients to treatment groups. An online random number generator (www.random.org—accessed between January 2012 and May 2015) was used by the nursing staff (circulating nurse), who was unaware of the study design and patient’s characteristics. The first group, the “bevacizumab group” consisted of eyes that had trabeculectomy with a single intracameral injection of 1.25 mg of bevacizumab while the “MMC group” consisted of eyes that underwent standard guarded trabeculectomy with intra-operative application of 0.02% MMC soaked sponges on bare sclera for 2 min under Tenon’s capsule.

This was an open-label study.

### 2.8. Sample Size and Statistical Analysis

Considering our own surgical outcomes in a historical cohort of patients, as well as success rates in studies with similar criteria [5,20], we performed a power analysis selecting an α-error of 5% and a power of 80%. Accordingly, 47 patients in each group would be required in order to detect a 20% difference in the rate of complete success, assuming a 25% failure rate in the MMC group at three years. We recruited 100 patients in total.

Statistical analysis was carried out with Microsoft Excel (Microsoft Corporation, Redmond, WA, USA) and NCSS 11.0 (NCSS, LLC., Kaysville, UT, USA) software. The Kolmogorov–Smirnov test was employed to confirm normal distribution of datasets. The two-tailed paired *t*-test and the unpaired *t*-test (Student’s *t*-test) were used to compare normally distributed, continuous paired and unpaired data, respectively. In cases of non-normally distributed continuous data, non-parametric tests were employed—the Wilcoxon Signed-rank test and the Mann–Whitney U test to compare paired and unpaired datasets, respectively. Fisher’s exact test was used to compare categorical variables. The log-rank test was used to compare Kaplan–Meier survival curves for both complete and qualified success between the two groups.

## 3. Results

**Figure 1:**
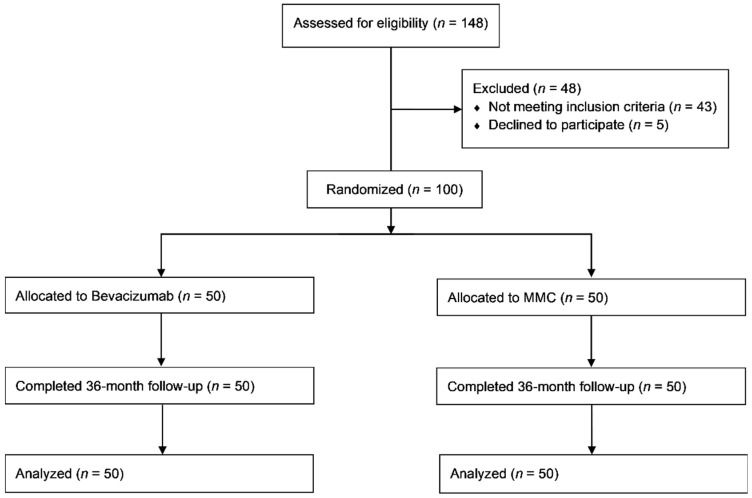
CONSORT flow diagram demonstrating the number of patients assessed, enrolled, randomized and analyzed. MMC: Mitomycin C.

A total of 148 patients were screened for eligibility between January 2012 and May 2015, of whom 100 patients met the eligibility criteria and were randomized. Fifty patients were assigned to the bevacizumab group and 50 patients to the MMC (control) group, all of them being Caucasian. All 100 patients completed the study, with no one being lost to follow-up (Figure 1). Demographics of the two groups with regards to the antimetabolite/antifibrotic agent used are summarized in Table 1. There was no statistically significant difference between the two study groups with respect to any of the reported parameters.

**Figure 2:**
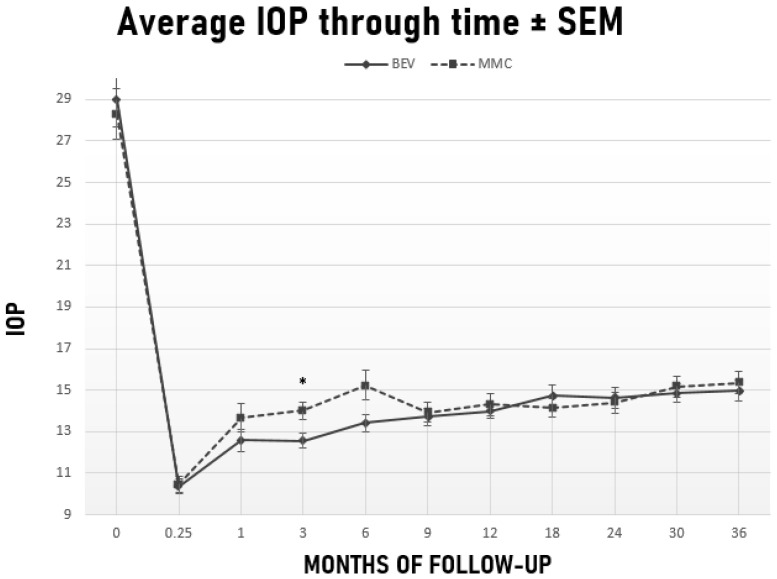
Course of IOP ± Standard Error of Measurement (SEM) for the Bevacizumab (BEV) and MMC cohorts, respectively. Error Bars represent standard error of measurement at respective time-points. * *p* < 0.05 Mann–Whitney U test.

Preoperative IOP improved significantly from 29 ± 9.4 mmHg to 15 ± 3.4 mmHg in the bevacizumab group (*p* < 0.001) and from 28.3 ± 8.7 mmHg to 15.4 ± 3.8 mmHg in the MMC group (*p* < 0.001) at 36 months (Figure 2). The number of glaucoma medications also decreased significantly from 3.5 ± 0.9 preoperatively to 0.5 ± 1 in the bevacizumab group (*p* < 0.001) and from 3.6 ± 0.7 to 0.6 ± 1.1 in the MMC group (*p* < 0.001) at 36 months. No significant difference was detected between the two groups in neither levels of IOP nor medication requirements at 36 months (*p* = 0.6; *p* = 0.7, respectively). Table 2 shows IOP levels and glaucoma medication requirements at each follow-up point. IOP was significantly lower in the bevacizumab group 3 months after surgery (IOP = 12.6 ± 2.5 mmHg, 95% CI, (11.9, 13.2) vs. 14 ± 3 mmHg, 95% CI, (13.2, 14.9), *p* = 0.01). The difference in IOP barely missed statistical significance 6 months after surgery in favor of the bevacizumab group (Table 2).

**Figure 3:**
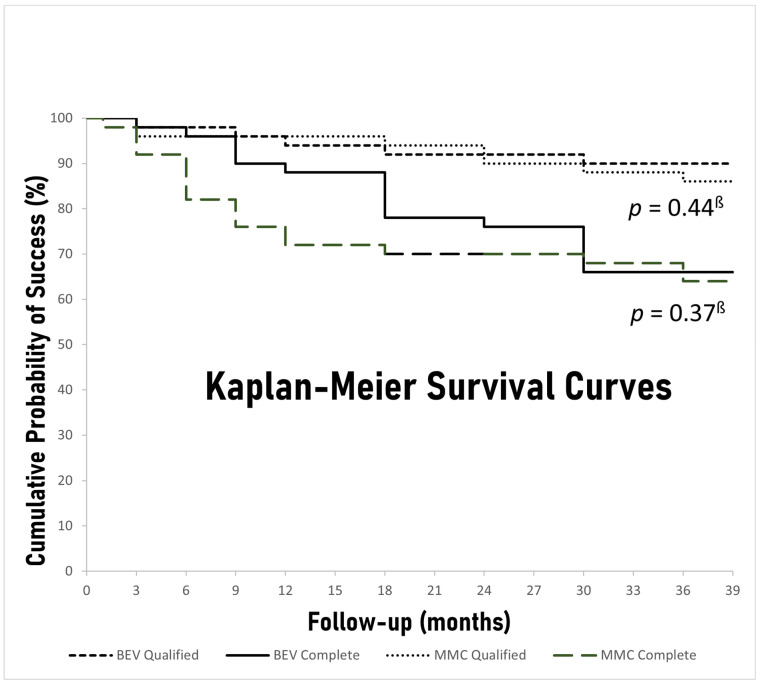
Kaplan–Meier survival curves for both complete and qualified success in the Bevacizumab (BEV) and Mitomycin-C (MMC) cohorts. ^ß^ log-rank chi-square test.

The rates of complete and qualified success during the predetermined time-points of follow-up are depicted in Table 3. There was no significant difference between the bevacizumab and MMC groups in either complete or qualified success rates at 36 months (66% vs. 64%, *p* = 0.50 and 90% vs. 86%, *p* = 0.38, respectively). Moreover, Kaplan–Meier survival curves of both complete and qualified success for both study groups were constructed, and the respective *p* values derived from log-rank chi-squares were calculated (Figure 3). No significant difference between the groups was discovered for either complete or qualified success when all the follow-up period was taken into account (*p* = 0.37, *p* = 0.44, respectively). Nevertheless, the bevacizumab group had significantly higher rates of complete success 6 and 12 months after surgery (96% vs. 82% *p* = 0.03, 88% vs. 72% *p* = 0.04, respectively) and barely missed statistical significance at 9 months (90% vs. 76% *p* = 0.05). Overall, 12 patients (12%), 5 (10%) in the bevacizumab group and 7 (14%) in the MMC group, failed to meet either complete or qualified success criteria at the last follow-up visit (Table 3). The most frequent reason for failure was IOP reduction less than 20% (*n* = 8), whereas, 1 patient (2%) in the bevacizumab group and 3 patients (6%) in the MMC group required reoperation for failing blebs at 29, 17, 30, and 34 months, respectively (Table 4).

**Figure 4:**
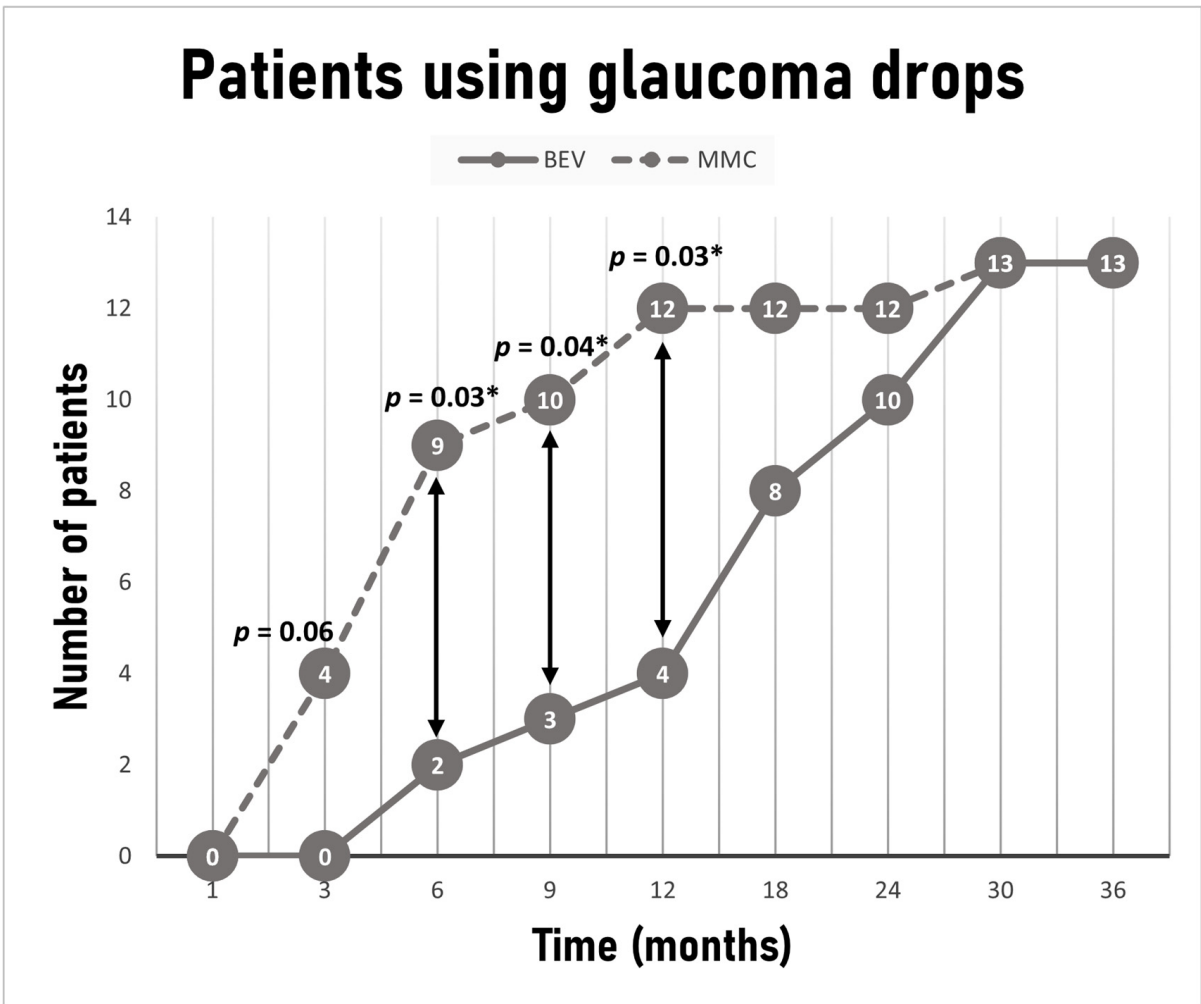
Total number of patients needing additional IOP lowering drops during the follow-up period. Time points reflect initiation of treatment. * *p* < 0.05 Fisher’s exact test.

Post-operative interventions were at the discretion of the treating physician and relied on subjective assessment of bleb characteristics such as thickness, elevation, encapsulation and vascularity and secondarily on IOP trends after surgery. All such interventions were performed during the first six post-operative months. Results are illustrated in Table 4. Although patients belonging to the MMC cohort required slightly more 5-FU injections compared to the bevacizumab cohort, the difference was insignificant (*p* = 0.12). Furthermore, a similar number of patients required needling between the two groups (*p* = 0.34). The decision to resume glaucoma medications after surgery was also at the discretion of the treating physician and relied on target IOP, as determined by age, severity of perimetric loss, CCT, diagnosis and pre-operative level of IOP, thought to have been associated with disease progression. Medications resumed in the following order provided tolerance and absence of contraindications: prostaglandin analogues, b-blockers, carbonic anhydrase inhibitors, a2-agonsits. Even though the same number of patients in each group was on IOP lowering drops at last visit (*n* = 13, 26% in each group), a statistically significant difference between the groups was noted at 6, 9 and 12 months, with fewer patients requiring medications in the bevacizumab group (2 (4%) vs. 9 (18%) *p* = 0.03, 3 (6%) vs. 10 (20%) *p* = 0.04, 4 (8%) vs. 12 (24%) *p* = 0.03, respectively) (Figure 4). The average time for initiation of treatment for patients requiring medications was 7.6 ± 5.8 months for the MMC group and 18.7 ± 8.7 months for the bevacizumab group (*p* < 0.001).

Postoperative complication rates are presented in Table 4. Although the MMC group had more complications, there was no significant difference between the two groups. We report no cases of persistent hypotony, i.e., IOP < 6 mmHg lasting more than 1 month, suprachoroidal hemorrhage, vitreous prolapse, malignant glaucoma or bleb-related endophthalmitis. There was one case of blebitis in the MMC group which happened 17 months after surgery and was managed conservatively.

## 4. Discussion

This study compares the results of trabeculectomy with a single intraoperative intracameral injection of bevacizumab to trabeculectomy with standard sub-Tenon’s application of MMC sponges. IOP course, medication requirements, the need for postoperative interventions to prevent bleb failure and complication rates after surgery were evaluated and compared along with survival rates utilizing widely accepted methodology. This prospective, randomized study reflects, to our knowledge, the longest reported follow-up period on this subject to date.

We were able to demonstrate at least similar efficacy and safety of intracameral administration of bevacizumab as an adjunct to trabeculectomy compared to traditional MMC application. In both groups, IOP and medication requirements decreased significantly after surgery. In fact, at the end of the 36-month follow-up period, both the bevacizumab and MMC groups achieved similar IOP percentage reduction (48% vs. 46%) with similar medication requirements. Moreover, the rates of complete and qualified success between the two groups were also similar at 3 years. Earlier on, however, and specifically between 3 and 12 months postoperatively, patients receiving intracameral bevacizumab were at an advantage in terms of surgical efficacy over those who received MMC. Bevacizumab-treated patients had significantly lower IOP at month 3, achieved significantly higher rates of complete success at months 6 and 12, and were in significantly less need of glaucoma medications at months 6, 9 and 12. In common practice, if target IOP is not achieved or IOP starts to rise after surgery, patients are started on IOP lowering drops. As a result, IOP and qualified success might not differ in our study, but this may occur at the expense of more medications and lower complete success rates. In this study, MMC-treated patients required glaucoma drops earlier compared to the bevacizumab-treated ones.

Off-label intraocular administration of bevacizumab has been proven safe in the management of macular edema of various etiology [22]. We preferred bevacizumab over other anti-VEGF molecules because of lower cost and larger molecular weight allowing binding to the sclera potentially for a longer period of time, possibly resulting in a longer-lasting effect [23]. We chose to inject bevacizumab intracamerally, as opposed to the subconjunctival and intravitreal routes of administration, since we believe that direct injection into the anterior chamber ensures diffuse and more homogeneous distribution of the drug to the bleb through the sclerostomy. Moreover, VEGF levels in the aqueous humor of glaucoma patients rise after glaucoma surgery [13]. Thus, intracameral administration of the drug might promote direct anti-VEGF action in the aqueous humor, resulting in lower VEGF levels reaching the bleb. Furthermore, unnecessary manipulations of the conjunctiva that might further stimulate fibrosis are avoided and, by using the readily available paracentesis, complications related to intravitreal injections are prevented. Finally, bevacizumab has been well documented to be safe for corneal endothelial cells [24,25].

Only a few studies have addressed intracameral administration of bevacizumab during glaucoma filtration surgery. Vandewalle et al. [18] and Fakhraie et al. [19] showed that bevacizumab significantly enhances the short-term success of trabeculectomy when compared to placebo. Vahedian et al. [20] concluded that bevacizumab and MMC are similar as adjuncts to trabeculectomy in an RCT with approximately 18 months follow-up. Our study is in accordance with the cited literature, but reflects twice as long follow-up. Furthermore, it demonstrates that bevacizumab leads to significantly less short-term glaucoma medication requirements compared to MMC. This could be attributed to the ability of bevacizumab to bind to the sclera, allowing prolonged release of the drug into the subconjunctival space [26], and therefore, longer wound healing modulation. In fact, we were intrigued to further investigate whether repeated administrations of intracameral bevacizumab could prolong this drop-free period. Unlike Fakhraie et al. [19] and Vahedian et al. [20] who reported increased risk of early bleb leaks after bevacizumab, our study had very low rates of early bleb leakage, similar for both groups. Transient early hypotony, reflecting overfiltration, was the most common complication and was self-limited in all instances.

Limitations of this study include the relatively small sample size and the fact that the principal investigator was not masked with respect to study group assignment, which however, would have been difficult to accomplish as the methodology of drug administration is markedly different in the two study groups. Larger sample sizes are required to precisely detect smaller than 20% differences in success rates. Furthermore, bleb morphology was not systematically evaluated nor quantified in this study. Longer follow-up and larger sample sizes are required to determine if the study groups differ with respect to late or uncommon complications, such as blebitis and bleb-related endophthalmitis. Bleb morphology and vascularity are directly linked to the incidence of such complications [27].

In conclusion, intracameral administration of bevacizumab during trabeculectomy results in blebs that function equally well at 3 years compared to those augmented with sub-Tenon’s MMC, and at the same time, significantly reduces the number of patients needing IOP lowering medications during the first year of follow-up. Thus, it can be considered as a substitute for MMC in glaucoma filtration surgery. Longer follow-up and larger sample sizes are required to further investigate this alternative.

## Figures and Tables

**Table 1:** Preoperative demographic characteristics for both study groups.

Preoperative Characteristics	Bevacizumab Group	MMC Group	*p* Value
Number of eyes included	50	50	-
Eyes with 36 months follow-up	50	50	-
Male/Female	27/23	25/25	NS ^§,7^
Mean Age ± SD ^1^ (years)	71 ± 12.6	70.1 ± 10.2	0.69 ^ß^
Mean IOP ^2^ ± SD (mmHg)	29 ± 9.4	28.3 ± 8.7	0.7 ^ß^
Mean medications ± SD	3.5 ± 0.9	3.6 ± 0.7	0.53 ^ß^
Mean MD ^3^ ± SD (dB)	−14.3 ± 9.3	−13.7 ± 8.9	0.74 ^ß^
Mean CCT ^4^ ± SD (μm)	536 ± 19	533 ± 19	0.54 ^ß^
Mean cup to disk ratio ± SD	0.71 ± 0.1	0.68 ± 0.1	0.67 ^ß^
Lens Status			
Pseudophakia, *n* (%)	10 (20%)	13 (26%)	0.32 ^§^
Type of Glaucoma			
POAG ^5^, *n* (%)	38 (76%)	36 (72%)	0.41 ^§^
XFG ^6^, *n* (%)	12 (24%)	14 (28%)	

^1^ SD: Standard Deviation. ^2^ IOP: Intraocular Pressure. ^3^ MD: Mean Deviation in Humphrey Visual Fields. ^4^ CCT: Central Corneal Thickness. ^5^ POAG: Primary Open-Angle Glaucoma. ^6^ XFG: Exfoliative Glaucoma. ^7^ NS: not significant. ^ß^ Student’s *t*-test. ^§^ Fisher’s exact test.

**Table 2:** Postoperative IOP levels and number of glaucoma medications at each follow-up visit. Medications added at any respective time-point are reflected at the next follow-up visit. ^ß^ Student’s *t*-test, ^‡^ Mann–Whitney U test.

Postoperative IOP Levels and Number of Medications	Bevacizumab Group	MMC Group	*p* Value
Mean IOP ± SD at 1 day (mmHg) Medications	9.4 ± 3.1 0.0 ± 0.0	9.7 ± 3.3 0.0 ± 0.0	0.73 ^ß^ -
Mean IOP ± SD at 1 week (mmHg) Medications	10.4 ± 2.5 0.0 ± 0.0	10.5 ± 2.7 0.0 ± 0.0	0.82 ^ß^ -
Mean IOP ± SD at 1 month (mmHg) Medications	12.6 ± 3.7 0.0 ± 0.0	13.7 ± 4.9 0.0 ± 0.0	0.5 ^‡^ -
Mean IOP ± SD at 3 months (mmHg) Medications	12.6 ± 2.5 0.0 ± 0.0	14.0 ± 3.0 0.0 ± 0.0	0.01 ^‡^ -
Mean IOP ± SD at 6 months (mmHg) Medications	13.4 ± 3.0 0.0 ± 0.0	15.2 ± 5.1 0.1 ± 0.4	0.08 ^‡^ 0.05 ^ß^
Mean IOP ± SD at 9 months (mmHg) Medications	13.7 ± 2.9 0.1 ± 0.4	13.9 ± 3.2 0.3 ± 0.7	0.72 ^ß^ 0.07 ^ß^
Mean IOP ± SD at 12 months (mmHg) Medications	14.0 ± 2.2 0.1 ± 0.5	14.3 ± 3.9 0.3 ± 0.7	0.61 ^ß^ 0.09 ^ß^
Mean IOP ± SD at 18 months (mmHg) Medications	14.8 ± 3.5 0.1 ± 0.5	14.1 ± 3.2 0.4 ± 0.8	0.34 ^ß^ 0.06 ^ß^
Mean IOP ± SD at 24 months (mmHg) Medications	14.6 ± 3.5 0.3 ± 0.7	14.4 ± 3.6 0.4 ± 0.8	0.74 ^ß^ 0.43 ^ß^
Mean IOP ± SD at 30 months (mmHg) Medications	14.9 ± 3.1 0.4 ± 0.9	15.2 ± 3.4 0.4 ± 0.8	0.65 ^ß^ 0.99 ^ß^
Mean IOP ± SD at 36 months (mmHg) Medications	15.0 ± 3.4 0.5 ± 1.0	15.4 ± 3.8 0.6 ± 1.1	0.60 ^ß^ 0.70 ^ß^

**Table 3:** Success rates at key time-points. ^§^ Fisher’s exact test.

Months of Follow-Up	Outcome Measures, *n* (%)	Bevacizumab *n* = 50	MMC *n* = 50	*p* Value
6 month	Complete Success	48 (96%)	41 (82%)	0.03 ^§^
	Qualified Success	49 (98%)	48 (96%)	0.50 ^§^
12 month	Complete Success	44 (88%)	36 (72%)	0.04 ^§^
	Qualified Success	47 (94%)	48 (96%)	0.50 ^§^
24 month	Complete Success	38 (76%)	35 (70%)	0.33 ^§^
	Qualified Success	46 (92%)	45 (90%)	0.50 ^§^
36m	Complete Success	33 (66%)	32 (64%)	0.50 ^§^
	Qualified Success	45 (90%)	43 (86%)	0.38 ^§^

**Table 4:** Postoperative interventions and complications during follow-up. ^§^ Fisher’s exact test.

Months of Follow-up	Interventions and Complications	Bevacizumab *n* = 50	MMC *n* = 50	*p* Value
0–6 month	Bleb Needlings	2 (4%)	3 (6%)	0.34 ^§^
0–6 month	Number of 5-FU injections	22	29	0.12 ^§^
0–6 month	Patients requiring 5-FU injections	10 (20%)	11 (22%)	0.69 ^§^
36 month	Patients on medications	13 (26%)	13 (26%)	1 ^§^
0–36 month	Choroidal Effusion	4 (8%)	7 (14%)	0.26 ^§^
0–36 month	Transient Hypotony <1 month	8 (16%)	9 (18%)	0.49 ^§^
0–36 month	Hyphema	1 (2%)	4 (8%)	0.18 ^§^
0–36 month	Shallow AC	5 (10%)	5 (10%)	1 ^§^
0–36 month	Early Bleb Leak	1 (2%)	2 (4%)	0.50 ^§^
0–36 month	Cataract Surgery	9/40 (22.5%)	11/37 (29.7%)	0.32 ^§^
0–36 month	Blebitis	0	1 (2%)	0.99 ^§^
17–34 month	Reoperation	1 (2%)	3 (6%)	0.31 ^§^

## Data Availability

The principal investigator, Gerasimos Kopsinis, had full access to all the data in the study and takes responsibility for the integrity of the data and the accuracy of the data analysis. The data presented in this study are available on request from the corresponding author.
